# Supplementary material for: Prediction and discrimination of skeletal muscle function by bioelectrical impedance vector analysis using a standing impedance analyzer in healthy Taiwanese adults
Source: PLoS One. 2020 Jun 12;15(6):e0231604. doi: 10.1371/journal.pone.0231604 (PMC7292401; doi:10.1371/journal.pone.0231604)
Supplement: Attachment — Submitted filename: rebutting letter_2020.5.6 (3).docx [file pone.0231604.s001.docx]

***Comments from reviewers:***

**Reviewer #1**

59 - Insert period.

**[Answer]:**

Thanks for pointing out the error. We have corrected the mistake as below:

(line 59)	and chronic disease may affect muscle mass and function [2]**.**

69 - Insert 'the' before general population.

**[Answer]:**

Thanks for pointing out the error. We have added the article “ the” before the noun as below:

(line 69)	In the general population, a lower HSG is……..

70-79 - Specific reported data from other studies is not necessary to include in the Introduction. This can be distracting to the reader. I suggest only including your study data reported in the Results section.

**[Answer]:**

Thanks for the suggestion. We have deleted line 70-79 in the revised manuscript. In addition, the rest of 2^nd^ paragraph was merged with the 1^st^ paragraph.

89 - Insert 'an' before alternating.

**[Answer]:**

Thanks for pointing out the error. We have corrected it in the revised manuscript as below:

(line 89)	properties such as resistance (R) and reactance (Xc) using **an** alternating current.

227-228 - Include parenthesis when describing 20% for group I and V, or not at all. Regardless, stay consistent.

**[Answer]:**

The sentence has been rephased as below:

(line 227-229)	The subjects were divided into 5 equal groups (group I to V) depending on their HGS level~~: group I (the highest 20%), and group II, III, IV, and V~~, with group V representing the lowest quintile~~20%~~.

283-284 - Which goodness of fit test was used.
**[Answer]:**

To make the sentence clear, we have change “goodness of fit” to R-squared values in the revised manuscript as below:

Compared to the regression model for HGS developed using inpatient subjects by Norman et al. [25], the independent variables included in our regression analysis for muscle function were the same, but our models exhibited better ~~goodness of fit~~**R-squared values** (r^2^ = 0.745-792) than theirs (r^2^ = 0.708).

**Reviewer #2**

With this study authors aimed to determine if bioelectrical impedance vector analysis assessment can predict muscle function in a specific healthy adult population. The methods used are appropriate but some details should be added to increase the completeness of the manuscript as well as additional discussion points that will aid in reader comprehension and interpretation.
Introduction should include a better justification of the study rationale and clarify the actual knowledge about bioelectrical impedance vector analysis to discriminate muscle function as well as BIVA assessment positions.
Discussion should include appraisal with other methodologies and results used in previous studies. It also should be discussed how the circumstances BIVA was tested (refrain from alcoholic beverages for at least 48 hours, avoid diuretics for 7 days fast for 4 hours prior to study, and not asses females during menstruation) can interfere with the results.

**[Answer]:**

We thank the reviewer for the suggestions. We have revised the introduction and discussion sections in accordance with the comments from the reviewer. The main changes in the introduction can be found in the last two paragraphs of then section.

The main reason for the subject preparation before BIVA such as refrain from alcoholic beverages for at least 48 hours, avoid diuretics for 7 days fast for 4 hours prior to study, and not asses females during menstruation is to have a good control of hydration status of the subject as variation in hydration status may affect the accuracy and precision of electric properties of biological tissues. We have done a literature review and summarized the methodologies in the end of the 2^nd^ paragraph in discussion as below:

However, BIVA approach still involves the use of raw bioelectrical impedance parameters and is sensitive to physiological changes associated with the tissue conductivity. Factors known to introduce variability in tissue electric properties include body position, hydration status, electrolyte concentration, exercise, skin temperature and phase of menstrual cycle [28, 44-46]. This study was performed to test the potential application of BIVA in assessing skeletal muscle function and had the advantage of testing on healthy subjects under a careful control of hydration status.

Also, we have included appraisal with the other BIVA methodologies and results in the 3^rd^ and 4^th^ paragraphs of discussion in the revised manuscript as below:

The most common application of bioelectric impedance measure is whole body mode, which measures the total body electrical parameters with electrodes placed on the ipsilateral arm and foot. Whole body BIVA result is well-known to correlate with many diseases, such as renal diseases, critically ill patients, obesity, sarcopenia, and cachexia [40, 41, 48-50]. However, whole body mode regards the body as a uniform cylinder and this assumption is not entirely correct as the human body has a complex shape [28]. Furthermore, this method cannot provide information about body segment individually, limiting its application.

Segmental body measure of bioelectric impedance is a less common application, which can be acquired by two methods. For the bioelectric impedance of the arm, segmental impedance components can be acquired using two pairs of electrodes attached to the ipsilateral hand and shoulder [50]. The distance between receiving electrodes is then used for adjusting the impedance parameters. This method has been used to assess the skeletal muscle of right arm in Alzheimer’s disease and healthy control, showing a lower phase angles and longer specific vectors in patients with Alzheimer’s disease [51]. However, this approach involves complicated process for electrode placement and is less practical in large scale study. Another segmental approach is proposed by Kushner R.F. [28] who made the assumptions that the human body is composed of five cylinders (two upper limb cylinders, one trunk cylinder and two lower lib cylinders) and tissue electric properties in each cylinder can be directly measured. For BIVA of the arm, impedance components are measured with electrodes placed on the ipsilateral arm and foot, and voltage electrodes on both hands [28, 52], as done in our study. With this method, the measured values of Xc and R are divided by standing height, which assumes a fixed proportion of limb length to body height. This approach is simple and convenient but less discussed.

Specific Comments:
Line
55 “Skeletal muscle is the largest organ in human body”: please review the statement and add the bibliographic reference

**[Answer]:**

We have added reference to the sentence as below:

(line 55)	Skeletal muscle is the largest organ in human body which accounts for 30% of body weight in women and greater than 40% of body weight in men [1,2].

References:

1.	Janssen I, Heymsfield SB, Wang ZM, Ross R. Skeletal muscle mass and distribution in 468 men and women aged 18-88 yr. J Appl Physiol (1985). 2000;89(1):81-8.

2.	Frontera W, Ochala J. Skeletal Muscle: A Brief Review of Structure and Function. Calcified tissue international. 2014;96.

59 “and chronic disease may affect muscle mass and function [2] Muscle mass can be indirectly”: add punctuation mark period

**[Answer]:**

Thanks for pointing out the error. We have corrected the mistake as below:

(line 59)	and chronic disease may affect muscle mass and function [2]**.**

62 “local muscle endurance [3].” Please verify the reference used

**[Answer]:**

Thanks for pointing out the error. We have used Endnote to cite and format the references and make sure their correctness and formatting. The correct references here were as below:

62 “local muscle endurance [5,6].”

5.	Jones DA, Rutherford OM, Parker DF. Physiological changes in skeletal muscle as a result of strength training. Q J Exp Physiol. 1989;74(3):233-56.

6.	Reid KF, Fielding RA. Skeletal muscle power: a critical determinant of physical functioning in older adults. Exerc Sport Sci Rev. 2012;40(1):4-12.

69 “In general population, a lower HSG is”: Correct HGS

**[Answer]:**

We have added “the” prior to general population as suggested by Reviewer 1. We have also done the corrections as below:

(line 69)	In **the** general population, a lower ~~HSG~~**HGS** .………..

(line 84)	, ~~HSG~~**HGS** has also been used to monitor.………..

(line 86)	, ~~HSG~~**HGS** is a more sensitive indicator to.………..

81, 82 “HGS is one of the recommended tools for assessing nutritional state [13].”: Consider altering to: HGS is one of the four recommended types of measurements to assess nutritional state. [13].

**[Answer]:**

We have revised the manuscript as suggested by the reviewer as below:

(line 81-82)	HGS is one of the **four** recommended **types of measurements**~~tools for~~ **to** assess~~ing~~ nutritional state.

226 “strsu3ength than R/H in all 3 modes.”: Change to: strength

**[Answer]:**

We have corrected the error spelling of strength in line 226.

258, 259 “Alternatively, segmental BIVA components may be measured with electrodes placed on the ipsilateral arm and foot, and voltage electrodes on both hands.”: Please add reference

**[Answer]:**

We have added references to the sentence below.

(line 258-259)	Alternatively, segmental BIVA components may be measured with electrodes placed on the ipsilateral arm and foot, and voltage electrodes on both hands [24, 46], as done in our study.

24.	Kushner RF. Bioelectrical impedance analysis: a review of principles and applications. J Am Coll Nutr. 1992;11(2):199-209.

46.	Organ LW, Bradham GB, Gore DT, Lozier SL. Segmental bioelectrical impedance analysis: theory and application of a new technique. J Appl Physiol (1985). 1994;77(1):98-112.

Table 1: verify legend

**[Answer]:**

Thanks for pointing out the error. We have corrected the error in the footnote as below:

HGS, hand grip strength; Z, impedance; R, resistance; Xc, reactance; R/H, resistance standardized for height; Xc/H, reactance standardized for height; PhA, phase angle; Lean, lean body mass; BF%, percentage body fat. Subscript DH, RH, LH, WB, RA, and LA denote dominant head, right hand, left hand, whole body, right arm and left arm, respectively.

Figure 1: add figure title and verify legend

**[Answer]:**

1.	Asselin MC, Kriemler S, Chettle DR, Webber CE, Bar-Or O, McNeill FE. Hydration status assessed by multi-frequency bioimpedance analysis. Appl Radiat Isot. 1998;49(5-6):495-7.

2.	Gudivaka R, Schoeller D, Kushner RF. Effect of skin temperature on multifrequency bioelectrical impedance analysis. J Appl Physiol (1985). 1996;81(2):838-45.

3.	Kushner RF. Bioelectrical impedance analysis: a review of principles and applications. J Am Coll Nutr. 1992;11(2):199-209.

4.	Gleichauf CN, Roe DA. The menstrual cycle’s effect on the reliability of bioimpedance measurements for assessing body composition. The American Journal of Clinical Nutrition. 1989;50(5):903-7.

For better demostration of Figure 1, we have revised the figure and legend as below:

Figure 1. Illustration of impedance measurement methods for the respective body parts.
