# Supplementary material for: Prediction and discrimination of skeletal muscle function by bioelectrical impedance vector analysis using a standing impedance analyzer in healthy Taiwanese adults
Source: PLoS One. 2020 Jun 12;15(6):e0231604. doi: 10.1371/journal.pone.0231604 (PMC7292401; doi:10.1371/journal.pone.0231604)
Supplement: Attachment — Submitted filename: rebutting letter_2020.5.31.docx [file pone.0231604.s002.docx]

***Comments from reviewers:***

**Reviewer #2**

Most of my concerns have been adequately addressed.
I ask you to review Line 55 “Skeletal muscle is the largest organ in human body which accounts for 30% of body weight in women and greater than 40% of body weight in men [1,2].” None of the cited references defines skeletal muscle an organ as it is a tissue so please change it accordingly.

**[Answer]:**

We thank the reviewer for her careful and professional review. We agreed with the reviewer that skeletal muscle is regarded as a tissue type instead of an organ in both original information as well as textbooks. Therefore, in our revised manuscript, the word “organ” has been replaced by “tissue”. Please see below for the change in the revised manuscript (lines 56-57):

Skeletal muscle is the largest ~~organ~~ tissue in human body which accounts for 30% of body weight in women and greater than 40% of body weight in men [1,2].
